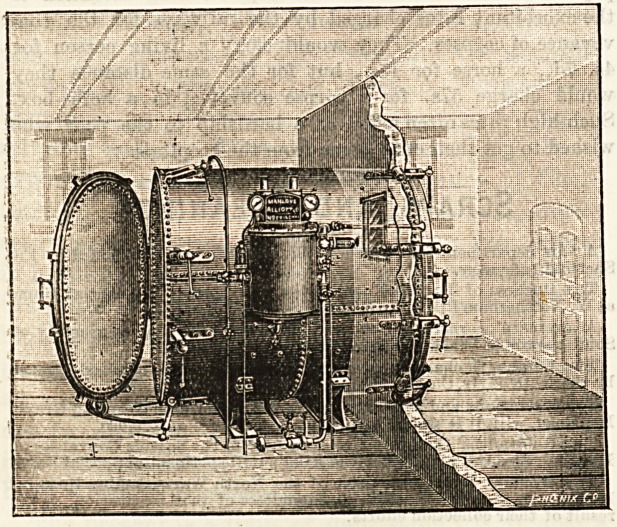# Steam Disinfector

**Published:** 1893-12-16

**Authors:** 


### PRACTICAL DEPARTMENTS.

### STEAM DISINFECTOR.

Messrs. Manlove, Alliott, and Co., of Nottingham, have recently brought out a disinfecting machine which has proved singularly successful in its workings, and seems likely to supersede those which have hitherto been in use. The apparatus which has been generally employed for disinfection by means of high pressure steam, the method which is universally admitted to be the best and most effectual, is that known as the "Washington Lyon's Patent Steam Disinfector." (A full description of this will be found in The Hospital for June 29th, 1893, page clxxiii.)

But though, when carefully attended to, this machine does its work well, yet it is still possible, should the doors be opened before the whole of the steam has been exhausted, for the contents to become moist and damp through the condensation thus caused. Messrs. Manlove and Alliott have endeavoured to overcome this drawback in their present invention, and would seem to have met with entire success, the object being "to secure the advantage of high pressure steam without the possibility of wetting the goods," and "to enable clothing, bedding, &c., to be thoroughly aired before leaving the machine." This end is effected by causing the air to be removed from the disinfecting chamber and the interstices of the infected articles by an air-pump or vacuum-producing apparatus, direct and thorough penetration of the goods by the warmed air or steam being thus absolutely assured.

Messrs. Manlove and Alliott's Disinfector consists of a large steam jacketted chamber, before entering which both steam and air are thoroughly dried and heated by passing through certain pipes or coils of solid drawn copper tubes, surrounded by hot steam from the jacket of the machine. The air thus admitted being subjected only to steam heat, cannot be raised to a higher temperature than that of the steam, and therefore no fear can be entertained of scorching the infected articles. This plan in actual practice is found to work well in every way, and clothing, bedding, &c., may thus be not only efficiently disinfected, but are dried and aired at the same time.

The machine is supplied with all necessary appliances for circulating steam or hot air where this method is preferred to that of merely filling the chamber. For the shape of these machines, the oval or elliptical form is found to be better in many respects than the circular or the rectangular. The latter is the least to be recommended of the three, being the weakest from a structural point of view. For the convenient reception of stiff and bulky articles such as mattresses, the oval is undoubtedly of the most practical value.

The steam for these disinfectors may be supplied in one of three ways—either by utilising steam from an existing boiler, by means of a special boiler made for the purpose, or by using the lower part of the apparatus itself as a steam boiler. The first of these methods is probably the best, where by any means attainable. Messrs. Manlove and Alliott have done a useful work by this excellent invention, as will be readily acknowledged by all who make a trial of its really great advantages. Our illustration is by kind permission of the firm.

## Figures and Tables

**Figure f1:**